# Mesocorticolimbic Dopamine Pathways Across Adolescence: Diversity in Development

**DOI:** 10.3389/fncir.2021.735625

**Published:** 2021-09-08

**Authors:** Lauren M. Reynolds, Cecilia Flores

**Affiliations:** ^1^Plasticité du Cerveau CNRS UMR8249, École supérieure de physique et de chimie industrielles de la Ville de Paris (ESPCI Paris), Paris, France; ^2^Neuroscience Paris Seine CNRS UMR 8246 INSERM U1130, Institut de Biologie Paris Seine, Sorbonne Université, Paris, France; ^3^Department of Psychiatry and Department of Neurology and Neurosurgery, McGill University, Douglas Mental Health University Institute, Montréal, QC, Canada

**Keywords:** dopamine, adolescence, development, mesocorticolimbic dopamine system, microglia, guidance cues, miRNA, puberty

## Abstract

Mesocorticolimbic dopamine circuity undergoes a protracted maturation during adolescent life. Stable adult levels of behavioral functioning in reward, motivational, and cognitive domains are established as these pathways are refined, however, their extended developmental window also leaves them vulnerable to perturbation by environmental factors. In this review, we highlight recent advances in understanding the mechanisms underlying dopamine pathway development in the adolescent brain, and how the environment influences these processes to establish or disrupt neurocircuit diversity. We further integrate these recent studies into the larger historical framework of anatomical and neurochemical changes occurring during adolescence in the mesocorticolimbic dopamine system. While dopamine neuron heterogeneity is increasingly appreciated at molecular, physiological, and anatomical levels, we suggest that a developmental facet may play a key role in establishing vulnerability or resilience to environmental stimuli and experience in distinct dopamine circuits, shifting the balance between healthy brain development and susceptibility to psychiatric disease.

## Introduction

Dopamine (DA) neurotransmission contributes to a multitude of behaviors, including motor control, reward learning, cognitive control, decision making, motivation, and salience attribution (Wise, 2004; Schultz, 2007; Bromberg-Martin et al., 2010; Cools and D’Esposito, 2011; Orsini et al., 2015; Coddington and Dudman, 2019). The ability of this modulatory neurotransmitter system to simultaneously direct different types of behavior may result from the heterogeneity of DA neurons originating in ventral midbrain nuclei and projecting to limbic or cortical regions. Limbic and cortical DA pathways have been shown to differ in their molecular markers, anatomical organization, and response to stimuli (Roeper, 2013; Lammel et al., 2014; Morales and Margolis, 2017; Poulin et al., 2018; Nguyen et al., 2021). However, less emphasis has been placed on the distinct developmental trajectories of dopamine projections. Mesocorticolimbic DA pathways continue to develop across postnatal life, throughout what is considered adolescence and, often, into early adulthood. Incorporating this knowledge in our understanding of DA diversity is particularly important since the DA system is increasingly considered as a “plasticity system,” whose development can be shaped by positive or negative experiences, allowing organisms to adapt to their surrounding environmental conditions (Barth et al., 2019; Reynolds and Flores, 2021).

**Figure 1:**
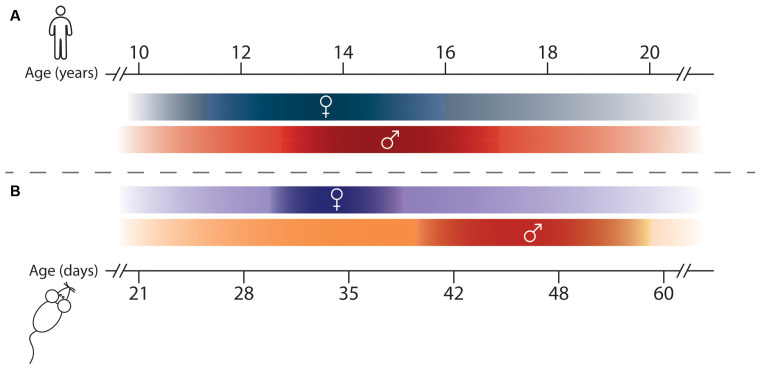
Adolescence and Puberty timing in humans and rodents. **(A)** Timeline of adolescence (shaded lines) and puberty (darker portions of the lines) in girls (♀) and boys (♂), adapted from Hollenstein and Lougheed (2013); Sawyer et al. (2018); and Brix et al. (2019). **(B)** Adolescence (shaded lines) and puberty (darker portions of the lines) timing in female (♀) and male (♂) rodents, adapted from Vetter-O’Hagen and Spear (2012) and Schneider (2013).

Adolescence is a time when organisms undergo dramatic physical, hormonal, and behavioral changes as they transition from juveniles to adults. While in humans this period has been historically framed as ranging from 12–20 years of age, the boundaries of adolescence are increasingly recognized as difficult to define precisely, with modern definitions extending between 10 and 24 years of age (Figure 1A; Hollenstein and Lougheed, 2013; Sawyer et al., 2018). The brain undergoes exuberant development during this time, with cortical gray matter thickness, notably in the prefrontal cortex (PFC), decreasing before stabilizing at adult levels, and with white matter volume increasing until early adulthood (Blakemore, 2012; Paquola et al., 2019). These macroscale changes likely result from cellular, molecular, and connectivity neuroadaptations in adolescence, as postmortem studies show dramatic age-dependent changes in myelination, neuronal structure, and synapse density during this time (Petanjek et al., 2011; Miller et al., 2012; Catts et al., 2013).

A particular interest in the postnatal development of DAergic systems has emerged because of evidence showing that performance in DA-dependent cognitive tasks improves gradually across adolescence (Wahlstrom et al., 2010; Luciana et al., 2012; Galvan, 2017; Larsen and Luna, 2018). However, direct evidence of cellular and molecular maturational changes in the human DA system across adolescence remains scarce, and it is limited to post-mortem studies which suggest that DA signaling remains dynamic across postnatal life (Weickert et al., 2007; Rothmond et al., 2012). Studies in adult volunteers can estimate DA release in subcortical regions by measuring the binding of radioactive ligands to DA receptors using positron emission tomography (PET). However, because of its radioactive nature, PET is contraindicated for imaging in minors without medical necessity (Ernst and Luciana, 2015). To overcome this impasse, recent studies have introduced tissue iron concentration as a proxy measure for DA concentration, as it is easily distinguishable using non-invasive functional magnetic resonance imaging (fMRI), can be extracted from existing fMRI datasets (Peterson et al., 2019), and is correlated with radioligand binding for the vesicular monoamine transporter (VMAT2) in adult subjects (Larsen et al., 2020). Tissue iron levels in the human striatum increase throughout adolescence, before stabilizing in adulthood (Larsen and Luna, 2014; Larsen et al., 2020), and are associated with the ongoing maturation of DA-dependent behaviors (Parr et al., 2021). While changes in tissue iron levels have not yet been assessed in the developing PFC, these results suggest that striatal DA regions are undergoing dynamic maturation in adolescence, mirroring previous findings from preclinical studies.

Most of our knowledge about adolescent mesocorticolimbic DA development comes from preclinical research, and from rodent studies in particular. One practical advantage of using rodents in developmental research is their compressed lifespan since they are born after about 3 weeks of gestation and reach adulthood at 2 months of age. When considering the age range of adolescence in rodents, it is important to keep in mind that while puberty and adolescence necessarily coincide temporally, these terms are not interchangeable (Spear, 2000; Schneider, 2013). Puberty can be clearly defined by the onset of sexual maturity, while adolescence is a more diffuse period representing the gradual transition from a juvenile state to independence. While studies in rodents consider a range of PND 28–42 to be *peri-pubertal* in male animals, *adolescence* is instead suggested to extend from the age of weaning (PND 21) until adulthood (PND 60; Spear, 2000; Tirelli et al., 2003; Burke and Miczek, 2013; Schneider, 2013; Figure 1B). This timeline encompasses the entire post-weaning period when rodents exhibit distinct neurobiological and behavioral changes and are navigating their environment independently for the first time. This demarcation is more aligned with the modern, extended definition of human adolescence (Sawyer et al., 2018).

In this review, we provide an overview of preclinical findings regarding the adolescent development of mesocorticolimbic DA pathways, by both situating it within a historical context and by emphasizing novel advances in understanding its cellular and molecular underpinnings. While the majority of preclinical work on DA development has been performed exclusively in males, we highlight throughout the review studies that include both males and female subjects, since sex differences have been noted in adult DA circuitry architecture and function. We outline proposed mechanisms by which adolescent experiences interact with developmental programming to shape adult mesocorticolimbic DA connectivity and function. Finally, we identify important gaps in our knowledge which present promising avenues for future research.

## Mesocorticolimbic Dopamine Circuit Organization

**Figure 2:**
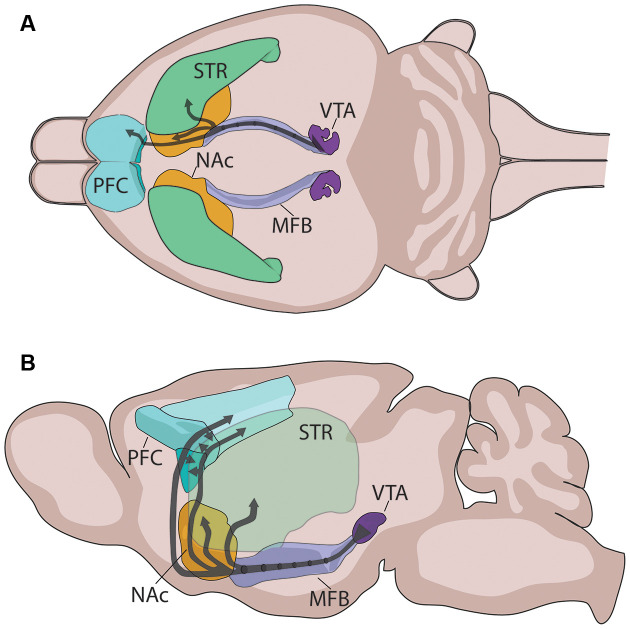
Mesocorticolimbic dopamine system organization. **(A)** Two-dimensional rendering of the mesocorticolimbic DA pathway (black) and the major nuclei of interest in the horizontal plane. **(B)** Sagittal view of the mesocorticolimbic DA pathway (black) and the major nuclei, with the STR semi-transparent in the foreground. Two-dimensional representations were adapted from the three-dimensional regions of interest in the Allen brain atlas (Wang et al., 2020). PFC, prefrontal cortex; NAc, nucleus accumbens; STR, dorsal striatum; MFB, medial forebrain bundle; VTA, ventral tegmental area.

Since its initial characterization in the 1960s, the anatomical organization of the rodent DA circuitry has been comprehensively described and reviewed on several occasions (a non-exhaustive list of reviews: Björklund and Lindvall, 1984; Björklund and Dunnett, 2007; Sesack and Grace, 2010; Yetnikoff et al., 2014a; Morales and Margolis, 2017). This review focuses specifically on the mesocorticolimbic DA circuitry, which consists of cell bodies located in the ventral tegmental area (VTA) that send ascending fibers rostrally toward limbic and cortical regions through the tightly fasciculated medial forebrain bundle (Figure 2A; Nieuwenhuys et al., 1982). At the level of the nucleus accumbens (NAc) these fibers diverge to reach their terminal target, with the densest innervation comprising mesolimbic DA axons that remain in the NAc or that extend to more dorsal regions of the striatum (STR). Mesocortical DA fibers course along the medial forebrain bundle with mesolimbic DA axons, but split off toward the PFC by either passing through the NAc, STR, and external capsule; or by extending ventrally to bypass the NAc before curving dorsally, just caudal to the olfactory bulb (Figure 2B; Kalsbeek et al., 1988, 1992; Voorn et al., 1988; Kolk et al., 2009; Manitt et al., 2011; Brignani and Pasterkamp, 2017). Interestingly, despite the close proximity of mesolimbic and mesocortical DA axons throughout their trajectory to forebrain regions, there is little or no overlap in the targets they innervate. Unlike other neuromodulatory systems, VTA DA neurons rarely send axon collaterals between different forebrain regions (Fallon, 1981; Fallon and Loughlin, 1982; Swanson, 1982; Lammel et al., 2008; Beier et al., 2015; Reynolds et al., 2018). Since mesocortical DA axons pass through the striatum *en route* to the PFC, the lack of mesocorticolimbic DA collaterals suggests that the striatum functions as a “choice point” (Stoeckli and Landmesser, 1998), where DA axons segregate into their cortical and limbic projections.

While these pathways begin to be established in embryonic or early postnatal development (see Riddle and Pollock, 2003; Prakash and Wurst, 2006; Heuvel and Pasterkamp, 2008; Money and Stanwood, 2013; Brignani and Pasterkamp, 2017 for detailed reviews), significant alterations in mesocorticolimbic DA wiring are increasingly observed in late postnatal development.

## Growth and Organization of The Mesocortical Dopamine Pathway in Adolescence

**Figure 3:**
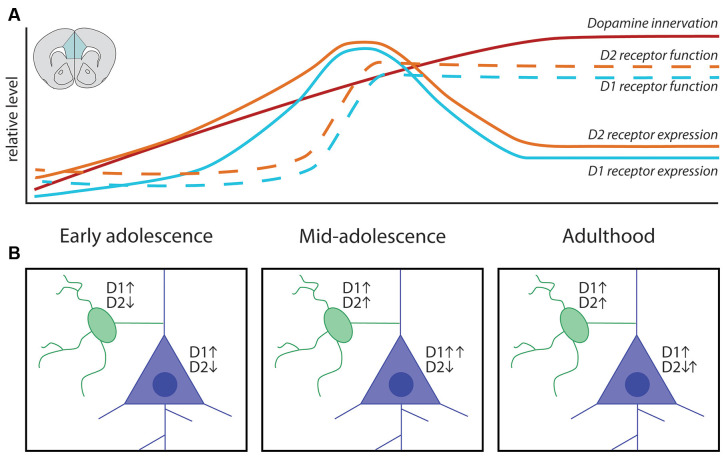
Adolescent maturation of dopamine connectivity and function in the prefrontal cortex. **(A)** Summary of maturational changes in DA connectivity in the PFC across adolescence. **(B)** Early in adolescence DA signaling through D1 receptors is excitatory onto both classes of neurons, with DA signaling through D2 receptors inhibiting pyramidal neurons and weakly inhibiting GABAergic interneurons. In mid-adolescence DA signaling through D2 receptors becomes excitatory onto GABAergic interneurons. DA signaling through D1 receptors is now able to interact with glutamatergic NMDA receptors on pyramidal neurons, increasing the activating effect of DA. In adulthood D1 and D2 receptor populations attain their mature function.

Reports that the density of mesocortical DA innervation continues to increase during adolescence were first published in the 1980s, shortly after the introduction of antibodies against tyrosine hydroxylase (TH, the rate-limiting enzyme of dopamine synthesis), which allowed clear detection of DA axons in the PFC (Verney et al., 1982). The earliest studies used light microscopy and TH immunofluorescence to show that DA axons in the supragenual anterior cingulate and prelimbic cortices of rats already show their typical thin morphology with irregularly spaced varicosities by early-to-mid adolescence. The density of DA fibers in these regions, however, continues to increase until PND 60 (Berger et al., 1985; Kalsbeek et al., 1988), with no further changes between PND 60 and PND 90 (Figure 3A). This adolescent increase in PFC DA innervation density remains a robust finding, as these initial qualitative descriptions have since been replicated (Benes et al., 1996) and extended by quantitative analysis in the PFC of rats and mice by several research teams (Manitt et al., 2011; Naneix et al., 2012; Willing et al., 2017; Hoops et al., 2018). Although the large majority of studies on mesocortical DA development have been performed only in male rodents, female rats have been shown to exhibit a similar pattern of innervation across postnatal ages (Willing et al., 2017).

The extended postnatal increase in PFC DA fiber density contrasts to other neuromodulatory systems, such as norepinephrine (NE) and serotonin, which reach adult PFC innervation density levels in rodents within the first 2–3 weeks of life (Levitt and Moore, 1979; Lidov et al., 1980). Distinguishing PFC DA axons from NE axons using immunolabeling for TH, which is required for the synthesis of both catecholamines, is often cited as a methodological concern for anatomical studies. Despite the fact that TH is also present in NE neurons, visually distinguishing DA and NE axons in the PFC is feasible. NE axons are thick, with regularly spaced rounded varicosities; while DA axons are thin and sinuous, with irregularly spaced varicosities (Berger et al., 1974; Miner et al., 2003). The two axonal populations differ further in their distribution, with DA fibers densely concentrated in the inner layers of the pregrenual and supragenual medial PFC, while NE fibers are spread across all layers (Berger et al., 1976; Levitt and Moore, 1979; Miner et al., 2003). Immunostaining for TH in the PFC labels PFC DA axons nearly exclusively because it only rarely overlaps with NE-specific markers, such as dopamine-β-hydroxylase (DBH) or the NE transporter (Pickel et al., 1975; Berger et al., 1983; Miner et al., 2003; Naneix et al., 2012). When compared directly within the same study, DA fiber density in the cingulate, prelimbic, and infralimbic subregions of the pregenual medial PFC has been shown to increase up to three-fold across adolescence, whereas DBH-stained NE fiber density remains stable across this time (Naneix et al., 2012). The seminal work of Rosenberg and Lewis shows that the same pattern of adolescent increase in PFC DA innervation is found in non-human primates, suggesting that this pattern is indeed conserved across mammalian species (Rosenberg and Lewis, 1994, 1995; Lewis, 1997).

**Figure 4:**
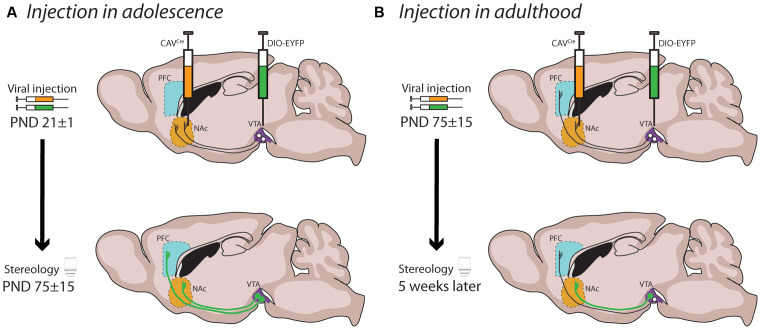
Mesocortical axon growth in adolescence revealed by an intersectional viral labeling technique. **(A)** Spatiotemporally specific labeling of DA neurons with axons present in the NAc at PND21 reveals that DA axons continue to grow to the PFC in adolescence. **(B)** Labeled DA axons are not found in the PFC of mice when subjected to the same manipulation in adulthood, supporting previous findings that DA neurons do not commonly send collaterals between these regions.

For many years this increase in PFC DA fiber density across adolescence was thought to represent the progressive increase in the sprouting of new branches from DA axons already innervating the PFC early in life, as long-range axon growth was assumed to be complete before adolescence. However, studies using anterograde or retrograde labeling have challenged this notion by suggesting that axons are still growing during postnatal development to connect from PFC to the amygdala (Arruda-Carvalho et al., 2017), or from the forebrain to the VTA (Yetnikoff et al., 2014c). By harnessing an intersectional viral labeling technique (Figure 4), we were able to restrict fluorescent labeling only to DA neurons with axons present in the NAc at PND 21. When the mice reached adulthood, we found that a subset of these DA axons in fact grew through the NAc to reach the medial or orbital PFC during adolescence (Figure 4A; Hoops et al., 2018; Reynolds et al., 2018). When we performed these same intersectional viral injections in adult mice, we observed very few, if any, labeled DA axons in the PFC (Figure 4B), in line with the lack of collaterals observed in previous studies (Fallon, 1981; Fallon and Loughlin, 1982; Lammel et al., 2008; Beier et al., 2015). This discovery is the first proof of long-range growth of axons in adolescence and explains why the mesocorticolimbic DA system is particularly vulnerable to adolescent experiences.

The size of DAergic varicosities in the PFC increases from ~1.2 μm at PND 20 to ~2.4 μm by PND 60 (Benes et al., 1996), and PFC DA concentration increases significantly during this time (Nomura et al., 1976; Leslie et al., 1991; Naneix et al., 2012). Varicosities are sites of DA synthesis, release, and re-uptake, and in the PFC at least 93% of them form functional synaptic contacts with local neurons (Séguéla et al., 1988). DA axons form synapses onto PFC glutamatergic pyramidal neurons; which represent the primary projection neurons from the PFC to other regions of the brain (Goldman-Rakic and Brown, 1982; Goldman-Rakic et al., 1992; Krimer et al., 1997; Carr et al., 1999; Carr and Sesack, 2000; Lambe et al., 2000), a phenomenon that is conserved in humans, non-human primates, and rodents. GABAergic interneurons, and in particular those that express parvalbumin (e.g., fast-spiking interneurons), also receive inputs from DA axons and express high levels of DA receptors (Verney et al., 1990; Benes et al., 1993, 2000; Sesack et al., 1995, 1998; Le Moine and Gaspar, 1998; Seamans and Yang, 2004; Glausier et al., 2009; Tritsch and Sabatini, 2012). Both DAergic synapses onto PFC pyramidal neurons and GABAergic interneurons have been reported to increase during adolescence. A study in rats shows that the number of DA appositions onto PFC GABAergic interneurons increases in adolescence (Benes et al., 1996). A study in non-human primates shows that the number of DAergic, but not serotonergic, appositions onto pyramidal neurons proliferates during adolescence, however, no changes in the number of DA oppositions onto GABA neurons were detected in this study (Lambe et al., 2000). These results suggest that the release of DA in the PFC evolves during adolescence in parallel to the establishment of mature pre-synaptic connectivity. Indeed, disruption of PFC DA innervation in adolescence results in altered dendritic arborization and dendritic spine density of layer V pyramidal neurons, indicating that PFC DA development in adolescence drives the structural maturation of local PFC circuits (Manitt et al., 2011, 2013; Reynolds et al., 2018).

## Postsynaptic Changes Across Adolescence in The Mesocortical Dopamine System

Both pyramidal and GABAergic interneurons in the PFC express DA receptors of the D1 (D1 and D5) and D2 (D2, D3, D4) families (Gaspar et al., 1995; Vincent et al., 1995; Knable and Weinberger, 1997; Lu et al., 1997; Davidoff and Benes, 1998; Le Moine and Gaspar, 1998; Mitrano et al., 2014). DA receptors are seven transmembrane G-protein coupled receptors that initiate intracellular cascades by increasing cAMP (D1-type) or decreasing cAMP (D2-type; Seamans and Yang, 2004; Tritsch and Sabatini, 2012). DA receptors are localized to apical and basilar dendritic arbors of pyramidal neurons and to dendrites and cell bodies of GABAergic interneurons that synapse onto pyramidal neurons. Signaling through PFC postsynaptic DA receptors thus directly and indirectly modulates PFC output (Gulledge and Jaffe, 2001; Seamans et al., 2001; Dong and White, 2003; Trantham-Davidson et al., 2004; Santana et al., 2009; Tritsch and Sabatini, 2012) and calibrates the balance of excitation/inhibition in the PFC, which also matures in adolescence (O’Donnell, 2011).

While the density of DA fibers in the PFC shows a linear increase across adolescence, the trajectory of postsynaptic DA receptor expression is more complex (Figure 3A). Autoradiography studies in rats using radio-labeled DA receptor ligands ([^3^H]SCH-23390 for D1-like receptors, [^3^H]nemonapride (YM-09151–2) or [^3^H]-raclopride for D2-like receptors) show discrepant results regarding the patterns of receptor expression across postnatal life. While some studies indicate a steady increase in PFC D1-like receptor density until PND 60 in rats (Tarazi et al., 1999; Tarazi and Baldessarini, 2000), others show that D1-like expression in the PFC peaks in adolescence, before being pruned back in adulthood (Leslie et al., 1991; Andersen et al., 2000; Brenhouse et al., 2008). These seemingly disparate findings may be reconciled by results from immunohistochemical tracing experiments showing that the adolescent peak in D1 receptor expression observed in adolescence occurs only in corticolimbic pyramidal projection neurons (Brenhouse et al., 2008; Brenhouse and Andersen, 2011), a level of nuance which may be difficult to capture with radioligand binding assays. Another consideration is that different subtypes of DA receptors may not follow the same developmental pattern of expression, but most radioligands do not differentiate between different receptors of the same family [e.g., [^3^H]SCH-23390 will bind both D1 and D5 DA receptors, which are expressed in the rodent PFC (Lidow et al., 2003)]. Recent results from quantitative real-time PCR (qPCR) studies in rats bolster the idea that PFC D1 and D5 receptor subtypes show a peak in mRNA levels during adolescence (Naneix et al., 2012; Zbukvic et al., 2017).

Regarding D2-like receptors, early radioligand studies in rats also indicate that their expression in the PFC increases steadily in adolescence (Tarazi et al., 1998; Tarazi and Baldessarini, 2000), but later findings show peak expression in adolescence (Andersen et al., 2000; Brenhouse and Andersen, 2011). This inconsistency may also stem from the non-specific nature of radioligand binding, as Naneix et al. (2012) show an adolescent peak in mRNA expression for the long isoform of the D2 receptor and the D4 receptor, but not for the short isoform of the D2 receptor, in the PFC using qPCR. Another consideration when assessing apparent discrepancies in the literature is that radioligand binding assays indirectly determine receptor protein levels and/or functional capacity, whereas qPCR determines mRNA expression. Overall, evidence from studies in rats indicates that DA receptor expression in the PFC is dynamic in adolescence, most likely with a period of overexpression followed by pruning (Figure 3A). However, this adolescent peak is less apparent at least in C57BL6 mice, according to an autoradiography study (Pokinko et al., 2017), suggesting differences across species. It should be noted that the aforementioned receptor expression studies were performed exclusively in male rodents and whether sex differences exist in the trajectory of PFC DA receptor expression remains an open question.

In addition to the dynamic changes in DA receptor expression, postsynaptic responses to extracellular DA have also been shown to evolve in PFC pyramidal and GABAergic neurons during adolescence (Figure 3B; O’Donnell, 2010, 2011). Slice electrophysiology experiments have shown that both pyramidal and GABAergic PFC neurons respond to DA application (Seamans and Yang, 2004). Studies in adult rats show that DA signaling through D1 receptors in PFC pyramidal neurons interacts with glutamatergic NMDA receptor signaling, producing activity levels that resemble those observed *in vivo* during information processing. Notably, this process is absent before mid-adolescence (Tseng and O’Donnell, 2005), emerging only around PND 45, when D1 mRNA expression peaks in the rat PFC (Tseng and O’Donnell, 2005; Naneix et al., 2012). In fact, before PND 36, DA signaling through D1 receptors in GABAergic interneurons potentiates their firing, but signaling through D2 receptors has either a weak *inhibitory* effect or no effect (Gorelova et al., 2002; Tseng and O’Donnell, 2007). However, after adolescence an *excitatory* effect of D2 receptor stimulation emerges in PFC GABAergic interneurons, creating the inhibitory tone characteristic of the mature PFC and balancing the enhanced DA-driven excitation of pyramidal neurons (Tseng and O’Donnell, 2007; O’Donnell, 2011). The adolescent shift of DAergic regulation over excitatory and inhibitory transmission in the PFC is thought to be a critical step in the developmental calibration of cognitive control (Klune et al., 2021), and to be dysregulated in psychiatric disorders of adolescent onset (O’Donnell, 2011; Caballero et al., 2016; Caballero et al., 2021).

## Coming of Age in The Striatum: Mesocorticolimbic Dopamine Pathway Segregation and Functional Maturation

As our understanding of mesocorticolimbic DA system development progresses, it is clear that both mesolimbic and mesocortical pathways are still developing throughout the adolescent period. DA signaling in the striatum continues to mature across adolescence: both TH protein and DA content increase until adulthood (Pardo et al., 1977; Giorgi et al., 1987; Broaddus and Bennett, 1990; Rao et al., 1991; Naneix et al., 2012; Matthews et al., 2013; Lieberman et al., 2018), and are essential for the construction of postsynaptic circuits. Medium spiny neurons (MSNs) in the NAc and STR achieve their namesake spiny appearance during early adolescence, with marked increases in dendritic spine density occurring between PND 15 and 30 (Tepper and Trent, 1993; Tepper et al., 1998). The proportion of MSNs showing their characteristic DA-sensitive inward rectification potassium currents also continues to increase until early adulthood (Tepper et al., 1998; Zhao et al., 2016), and the effect of D2 receptor signaling of MSNs switches from inhibitory to facilitatory (Benoit-Marand and O’Donnell, 2008). During this same timeline, NAc DA varicosities shift their synaptic contacts from the soma of MSNs to their dendritic spines (Antonopoulos et al., 2002), and the intrinsic excitability of MSN changes from a juvenile hyper-excitable state to a reduced, mature level of responsivity. This process is triggered by the gradual increase in striatal DA concentration (Lieberman et al., 2018).

**Figure 5:**
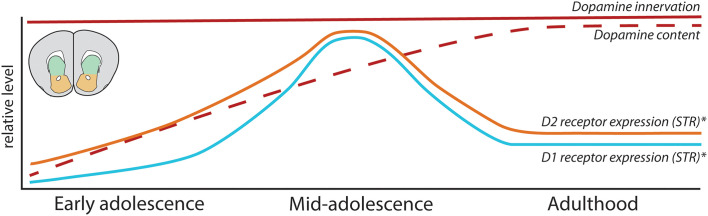
Development of mesolimbic dopamine connectivity and function in the adolescent striatum. Summary of maturational changes in DA connectivity in the STR across adolescence. *Indicates that these findings are sex-dependent, with this pattern of receptor overexpression and pruning only seen in male rodents.

In contrast to the PFC, the changes in DA function in the striatum are not associated with changes in the density of DA innervation (Figure 5), as STR and NAc DA input achieves its adult density by PND 20 in rodents (Voorn et al., 1988; Kalsbeek et al., 1992), and the optical density of TH-positive DA fibers does not change during adolescence in these regions (Naneix et al., 2012). Nevertheless, these results should be interpreted with care, considering that the DA innervation to the STR and NAc is up to 40-fold denser than in the PFC. It is possible that the margin of error, even using precise stereological methods, masks potential anatomical differences between ages (Bérubé-Carrière et al., 2012; Manitt et al., 2013; Reynolds et al., 2015).

Our developmental studies using intersectional viral tracing techniques indeed demonstrate that the fine organization of mesolimbic DA connectivity is much more malleable than previously thought. The striatum represents a major choice point for DA axons: while the large majority of DA axons already innervating the striatum by early adolescence are destined to remain there, mesocortical axons must pass through this densely innervated DA region and continue to grow into the PFC during adolescence (Reynolds et al., 2018). Notably, the level of DA innervation in these two pathways is inversely correlated: the more DA axons that keep growing to the PFC in adolescence, the fewer that remain behind and form connections in the NAc. Mesolimbic DA axon targeting is not a passive process [i.e., they are actively undergoing target recognition processes within the NAc in adolescence (Reynolds et al., 2018; Cuesta et al., 2020)], and it profoundly influences the structural organization of postsynaptic MSN neurons.

## Developmental Patterns of Dopamine Receptors in The Adolescent Striatum

In contrast to the high synaptic incidence of DA varicosities in the PFC (Séguéla et al., 1988), the majority of DA varicosities in the NAc and STR (60–70%) do not form direct synaptic contacts with postsynaptic neurons (Descarries et al., 1996; Descarries and Mechawar, 2000; Bérubé-Carrière et al., 2012). Recent reports further indicate that only ~30% of DA varicosities in the STR contain the necessary active zone sites to release DA, and that many varicosities are in fact “silent” (Pereira et al., 2016; Liu et al., 2018, 2021; Liu and Kaeser, 2019). Up to 90% of local striatal neurons are GABAergic projection neurons, usually referred to as MSNs or as spiny projection neurons (Kreitzer, 2009; Collins and Saunders, 2020), and they can be segregated into two main populations based on their projection target. In rodents, striatonigral MSNs project mainly to the substantia nigra pars reticulata and entopeduncular nucleus, while striatopallidal MSNs instead project primarily to the globus pallidus (Smith et al., 1998). While striatonigral and striatopallidal MSNs are morphologically indistinguishable at the somatic level, they can be differentiated by a number of molecular markers, notably by prominent expression of D1 receptors in striatonigral MSNs and D2 receptors in striatopallidal MSNs (Ince et al., 1997; Smith et al., 1998; Kreitzer, 2009; Bamford et al., 2018). Unlike the PFC, where ~25% of non-pyramidal neurons co-express D1 and D2 DA receptors (Vincent et al., 1995), D1 and D2 receptor expression is almost completely segregated between these two MSN populations (Hersch et al., 1995; Ince et al., 1997; Bertran-Gonzalez et al., 2010; Frederick et al., 2015). Interestingly, D1 and D2 colocalization in MSNs is apparent in embryos and neonates, but it decreases between E18 and PND 14 (Thibault et al., 2013; Biezonski et al., 2015). It remains to be determined whether the segregation of DA receptors in striatonigral and striatopallidal MSNs is dynamic at other postanal periods, as well as the age when the segregated pattern of MSN DA receptor expression is stabilized.

Rodent studies have shown that striatal DA receptor expression changes across adolescence, although their exact maturational pattern is controversial. Early autoradiography studies using the radioligand [^3^H]SCH-23390 to assess D1 receptor binding in Sprague–Dawley rats found that striatal D1 receptors either increase until achieving stable adult levels before or during early adolescence (Murrin and Zeng, 1990; Leslie et al., 1991; Schambra et al., 1994) or exhibit no developmental changes (Broaddus and Bennett, 1990). Instead, more recent studies using the same radioligand and rat strain show that, similarly to the PFC, DA D1-like receptors are overexpressed during adolescence before being pruned back in adulthood in the NAc and STR (Gelbard et al., 1989; Teicher et al., 1995; Andersen et al., 1997; Tarazi et al., 1999; Tarazi and Baldessarini, 2000). Similar findings using autoradiography have been reported in C57BL/6 mice (Pokinko et al., 2017).

Striatal expression of D2 receptors has also been reported to change during adolescence. Early autoradiography studies showed increased D2 receptor expression across early postnatal life, reaching adult levels by early adolescence (Pardo et al., 1977; Hartley and Seeman, 1983; Murrin and Wanyun, 1986; Rao et al., 1991; Schambra et al., 1994). This evidence seems to be consistent despite the use of different radioligands and strains of rats, with changes in expression in the NAc showing a less pronounced peak than in the STR (Teicher et al., 1995; Andersen et al., 1997; Tarazi et al., 1998), and mRNA expression of D2 receptors peaks in the adolescent STR (Naneix et al., 2012). These D2 expression changes have not been detected in mice (Pokinko et al., 2017).

While the majority of these studies have been performed only in male rodents, evidence suggests that the adolescent overexpression and pruning of D1 receptors in striatal regions is not seen in female rats (Andersen et al., 1997). Similarly, the adolescent overexpression and subsequent pruning in striatal D2 receptors observed in male rats is absent in females (Andersen et al., 1997), with no apparent sex differences in D2 receptor expression levels in adulthood. Males and females may therefore have distinct developmental trajectories for DA receptors in striatal regions, which could lead to different sensitive periods of development.

## Mechanisms Underlying Mesocorticolimbic Dopamine Circuit Organization in Adolescence

DA circuitry is increasingly recognized as a “plasticity system” (Barth et al., 2019; Reynolds and Flores, 2021), where the environment can alter its development and induce long-term repercussions for adult behavioral functioning. The protracted maturational timeline of mesocortical DA circuitry, therefore, results in a prolonged period of vulnerability, when experiences such as exposure to stress or drugs of abuse can disrupt its development and induce susceptibility to psychiatric disease later in life (Meaney et al., 2002; Gulley and Juraska, 2013; Jordan and Andersen, 2017; Areal and Blakely, 2020). The studies discussed in the preceding sections provide a holistic understanding of the developmental changes occurring in the mesocorticolimbic DA circuitry during adolescence but only recently have the cellular and molecular mechanisms underlying these processes begun to be unraveled. Below, we outline three main mechanisms identified to date which orchestrate adolescent mesocorticolimbic DA development and show examples of how they can be impacted by ongoing experiences.

### Role of Guidance Cues in Dopamine Axon Growth and Targeting

Guidance cues are secreted proteins, either diffusible or bound to cellular membranes, that act as a signal to direct growing axons to their appropriate targets (Battum et al., 2015). Their role in early DA development has long been appreciated, with a number of guidance cues shown to be implicated in the differentiation, migration, and early axonal pathfinding of DA neurons in embryonic and early postnatal life (see Heuvel and Pasterkamp, 2008; Bodea and Blaess, 2015; Brignani and Pasterkamp, 2017 for exhaustive reviews on the role of guidance cues in early DA development). The guidance cue Netrin-1 and its receptor DCC (deleted in colorectal cancer) have emerged as critical players in establishing mesocorticolimbic DA circuitry and are highly linked to psychiatric disorders of adolescent onset (Vosberg et al., 2019; Torres-Berrío et al., 2020a). Mice with *Dcc* haploinsufficiency show marked functional changes in DA systems, including blunted behavioral responses to amphetamine, methamphetamine, and cocaine (Flores et al., 2005; Grant et al., 2007; Flores, 2011; Kim et al., 2013; Reynolds et al., 2016); and blunted stimulant-induced DA release in the NAc (Grant et al., 2007). This protective *Dcc* haploinsufficient phenotype, which is also displayed by mice haploinsufficient for *Netrin-1*, is driven by increased DA innervation and content in the PFC, and by augmented stimulant drug-induced DA release in this region (Grant et al., 2007; Pokinko et al., 2015), indicating increased mesocortical inhibitory control over striatal DA function. Notably, these DAergic changes are only apparent in adult *Dcc* haploinsufficient mice, with no observable differences in mesocorticolimbic DA structure or function in juveniles, and occur in both males and females (Grant et al., 2009).

Netrin-1 and DCC are expressed across the lifetime in mesocorticolimbic DA circuits, and their spatiotemporal distribution is pathway-specific. DA neurons express high levels of DCC receptors across species, including humans (Osborne et al., 2005; Manitt et al., 2010; Reyes et al., 2013). Netrin-1 is expressed in forebrain terminal regions of DA axons, including the NAc, STR, and PFC (Shatzmiller et al., 2008; Manitt et al., 2011). In male rodents, the expression of DCC in the VTA and of Netrin-1 in the NAc is highest during embryonic and early postnatal development, waning gradually during adolescence, and stabilizing to low levels in adulthood (Manitt et al., 2010; Cuesta et al., 2018, 2020). While all mesolimbic DA axons are rich in DCC receptor levels, DA axons in the PFC only rarely express DCC (Manitt et al., 2011). The localization of DCC receptors in PFC DA axons is increased in adult *Dcc* haploinsufficent mice, suggesting that the greater PFC DA innervation and content results from ectopic growth of DCC-expressing mesolimbic DA fibers (Manitt et al., 2011). Conditional reduction of *Dcc* in DA neurons in adolescence recapitulates completely this ectopic DCC-positive DA axon phenotype in the PFC (Manitt et al., 2013).

Using the same intersectional viral labeling technique we used to demonstrate that mesocortical DA axons continue to grow to the PFC in adolescence, we also showed that the complementary action of Netrin-1 and DCC mediates the targeting of mesolimbic DA neurons at the NAc choice point. Reduced *Dcc* expression in DA axons innervating the NAc in adolescence, results in their ectopic growth in the PFC and a concomitant reduction in NAc DA varicosities (Reynolds et al., 2018). High levels of DCC in mesolimbic DA axons are necessary for them to recognize the NAc as their final target in adolescence. This phenotype is replicated when silencing Netrin-1 in the NAc (Cuesta et al., 2020).

Experience-induced regulation of Netrin-1 and/or DCC expression robustly shapes the adolescent brain. Social defeat stress in adolescence, but not in adulthood, downregulates *Dcc* expression in the VTA of male mice, disrupts PFC DA innervation, and leads to cognitive control deficits in adulthood (Vassilev et al., 2021). Mild traumatic brain injury in mid-adolescent male mice reduces Netrin-1 expression in the NAc and alters mesocorticolimbic DA organization (Kaukas et al., 2020). Both Netrin-1 and DCC levels expression can encode the effects of experience on DA circuitry. Notably, repeated exposure to amphetamine downregulates DCC in the VTA and Netrin-1 in the NAc, in early adolescence (Yetnikoff et al., 2007, 2011, 2014b; Cuesta et al., 2018, 2019), overlapping with the period that mesolimbic DA axons are undergoing targeting events. Drug-induced DCC downregulation requires D2 receptor signaling (Cuesta et al., 2018), reinforcing the link between DCC function and mesolimbic DA axon targeting in adolescence, as mesocortical DA neurons lack D2 receptors (Lammel et al., 2008). Exposure to recreational-like doses of amphetamine in early adolescence, but not later in life, leads to a dramatic increase in the volume of PFC DA innervation, altered DA function, and long-term impairments in cognitive control in male mice (Reynolds et al., 2015, 2019; Hoops et al., 2018; Reynolds and Flores, 2019). These effects are not observed following exposure to therapeutic-like amphetamine doses, which instead increases DCC protein expression in the VTA and leads to the overall improvement in cognitive performance in adulthood (Cuesta et al., 2019), in line with reports in non-human primates (Soto et al., 2012). Studies of how experience regulates Netrin-1/DCC expression in female mice are ongoing, but their bidirectional regulation already observed in male mice indicates that the Netrin-1/DCC system can be viewed more as a molecular target of *plasticity* rather than a target of *vulnerability*. Experiences that upregulate DCC expression in adolescence may in fact promote healthy brain development.

### Pruning of Connections in Adolescence: Microglia as Sculptors of Dopamine Circuitry

Neuro-immune interactions in adolescence are increasingly recognized as critical to the refinement of neural networks, and early immune challenges are a potential risk factor for DA-dependent neuropsychiatric disorders (Brenhouse and Schwarz, 2016). Microglia, in particular, have emerged as potent regulators of maturational processes, including activity-dependent synaptic pruning (Paolicelli et al., 2011; Schafer et al., 2012), the establishment of synaptic transmission and correlated brain activity (Zhan et al., 2014), and myelination in adolescence (Hughes and Appel, 2020). The neuroimmune system is tightly linked to the development of DA circuitry. Altered microglia function has been linked to DA system impairments, for example, DA damage in Parkinson’s disease patients (Ouchi et al., 2005) and D1 receptor deficiency in the PFC of adult ADHD patients (Yokokura et al., 2020). Changes in the expression of complement cascade proteins, important markers for immune-mediated phagocytosis and elimination, have been observed in schizophrenia patients (Sekar et al., 2016; Rey et al., 2020). Functional studies suggest these complement cascade alterations result in exaggerated synapse pruning in the adolescent PFC by overactive microglia (Sellgren et al., 2019) and in impaired social behavior in adulthood (Comer et al., 2020; Yilmaz et al., 2021).

Preclinical studies have further elaborated the role of microglia in normative mesocorticolimbic DA adolescent development. In the PFC, microglia transiently prune dendritic spines of the densely DA-innervated layer V PFC neurons in mid-adolescence (Mallya et al., 2018) and microglial depletion in adolescence impairs the formation and elimination of synapses onto these pyramidal neurons (Parkhurst et al., 2013). In the NAc, microglia play an important role in the elimination of DA receptors as Kopec et al. show that the peak in DA D1 receptor levels observed in the NAc around PND 30 in male rats declines afterward due to microglia pruning. In agreement with earlier autoradiography results (Andersen et al., 1997), this event occurs only in males and aligns with a peak in their social behavior. Females show an earlier peak (~PND20) in D1 receptor levels, which is followed by a microglia-*independent* decline in expression (Kopec et al., 2018). Indeed, a growing body of work shows that microglial processes are sex-dependent (Schwarz and Bilbo, 2012; VanRyzin et al., 2018, 2020; Bordt et al., 2020).

Several studies have linked experiences in adolescence to microglial changes within the mesocorticolimbic DA circuitry. Adolescent food restriction increases the ramification of microglia in the PFC of male and female rats (Ganguly et al., 2018), while social defeat stress decreases the number of PFC microglia in male mice and induced deficits in DA-dependent cognitive behavior (Reynolds et al., 2018; Zhang et al., 2019). Drugs of abuse in adolescence have been shown to induce noticeable changes in microglia expression in the NAc: nicotine exposure increases microglia ramification in the NAc of male and female mice in a DA D2 receptor-mediated process, leading to excessive synaptic pruning and increased cocaine self-administration in adulthood (Linker et al., 2020). Adolescent morphine exposure induces long-term changes in NAc microglial function in male rats, which are associated with increased conditioned place preference reinstatement to this drug in adulthood (Schwarz and Bilbo, 2013). Traumatic brain injury in adolescent, but not adult, mice increased microglia specifically in the NAc during early adulthood (Cannella et al., 2020), with a concomitant decrease in DA receptor expression. All of these findings poise microglial-mediated processes as a critical mechanism by which adolescent experiences shape mesocorticolimbic DA development.

### Puberty as a Driver of Dopamine Circuitry Development

Sex differences have been described regarding the structure and function of adult pre- and postsynaptic components of DA circuitries, including differences in structural organization, DA content, and regulation of local DA release (Becker et al., 2001, 2012; Becker, 2009; Gillies et al., 2014; Walker et al., 2017; Becker and Chartoff, 2018; Kokane and Perrotti, 2020; Zachry et al., 2020). Ovarian hormones are key regulators of some of these observed sex differences, notably DA neuron firing rates (Zhang et al., 2008; Calipari et al., 2017) and striatal DA release (Xiao and Becker, 1994; Castner et al., 2005; Calipari et al., 2017; Yoest et al., 2019). A greater number of DA neurons have been shown to project to the PFC in adult female rats in comparison to adult males, with ~50% of retrogradely labeled VTA neurons expressing TH in females compared to only ~30% in males (Kritzer and Creutz, 2008).

Because gonadectomy in adult animals alters PFC DA fiber distribution (Kritzer and Kohama, 1998; Kritzer, 1998, 2003; Adler et al., 1999; Kritzer et al., 1999), the pubertal spike in sex hormones has been long posited to drive sex differences in adult PFC DA innervation. However, contrary to the maturation of other PFC neurotransmitter systems (Drzewiecki et al., 2016, 2020; Piekarski et al., 2017; Delevich et al., 2020, 2021), evidence regarding a role for puberty in the development of mesocortical DA circuitry remains elusive. DA innervation to the PFC has been shown to increase along a similar timescale throughout adolescence in both male and female rats, with no apparent effect of puberty onset in this trajectory (Willing et al., 2017). However, puberty may drive subtle changes in PFC DA synthesis and release which would still profoundly impact the developing PFC, even without discernible alterations in DA axon architecture. For example, sex differences in PFC TH expression and in PFC neuronal organization emerge after puberty in mice with genetic reduction of the catechol-*o*-methyltransferase (COMT) enzyme (Sannino et al., 2017).

In the striatum, there are sex differences in the distribution of DA receptors, with female rats generally showing approximately 10% less D1 receptor density than males, and with D1 receptor density in females varying during the estrous cycle (Lévesque and Paolo, 1989, 1990; Lévesque et al., 1989). However, peripubertal sex hormones do not seem to play a role in establishing these sex-specific DA receptor patterns (Andersen et al., 2002). Sex differences in striatal DAergic structure and function have recently been suggested to be strain-dependent, with some of the sex-specific characteristics commonly seen in Sprague-Dawley rats not observable in Long-Evans rats (Rivera-Garcia et al., 2020), highlighting the need for the consideration not only of sex but also species and strain in experimental design. More evidence is needed to determine whether the sex differences observed in adult DA circuitry result from puberty-dependent or puberty-independent developmental processes. This issue will become clearer as SABV (sex as a biological variable) is increasingly included in study designs (Shansky and Murphy, 2021).

## Discussion

Aberrant mesocorticolimbic DA function is a prominent characteristic of psychiatric disorders that have an adolescent onset. Identification of the mechanisms underlying the normative maturation of this system during adolescence is essential to understand the developmental origins of mental health. The structure and function of pre- and postsynaptic components of mesocorticolimbic DA circuits differ significantly across terminal regions, between sexes, and as a function of experience. These differences include structural divergence, fluctuations in DA release, and/or variation in DA-induced modulation of postsynaptic neuron signaling pathways. Adolescence is a particularly sensitive time for the establishment of these properties; the discussion of DA projection heterogeneity is thus incomplete without considering the developmental programming of these systems.

While this review mainly focuses on advances in preclinical research, it is important to note that similar protracted developmental patterns in mesocorticolimbic DA circuitries have been observed in humans. As seen in rodents, post-mortem human brain studies have shown changes in DA receptor expression and DA content during adolescence, including a peak in DA D1 receptor expression in the PFC (Weickert et al., 2007; Rothmond et al., 2012), a marked decline in striatal DA receptors (Seeman et al., 1987), and a dramatic increase in striatal DA content (Haycock et al., 2003). A PET neuroimaging study in 18–32-year-old subjects shows that the decline in striatal D2/3 receptor expression also occurs in humans (Larsen et al., 2020), indicating that findings from preclinical studies on DA system development have strong translational implications.

**Figure 6:**
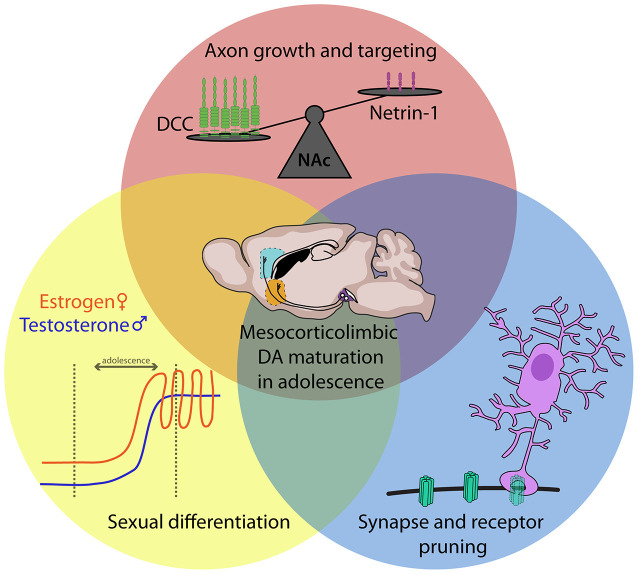
Dopamine as a “plasticity system”: convergent mechanistic processes shape adolescent mesocorticolimbic dopamine maturation in response to environmental cues. In this review, we identify three mechanistic processes that contribute to the adolescent establishment of DA pathways. We comment on how each of these processes may link experiential factors to alterations in DA development.

Here, we integrate evidence regarding mechanistic processes underlying mesocorticolimbic DA development while situating them within the historical context. We chose to highlight three mechanisms involved in DA maturation: axon guidance and targeting, microglial-dependent pruning, and puberty. By no means do we intend to imply that these are the only ongoing processes involved in mesocorticolimbic DA development. For example, macroautophagy has been suggested to play a role in the synaptic pruning occurring in the adolescent striatum (Hernandez et al., 2012; Lieberman et al., 2020). DA neurons projecting to the PFC have been shown to have different molecular properties than those projecting to the NAc (Lammel et al., 2008), suggesting that intrinsic differences may also contribute to their divergent development. Inputs to the VTA are also still developing in adolescence (Yetnikoff et al., 2014c), which may influence the maturation of DA neurons themselves. Indeed, *in vivo* electrophysiology experiments indicate that DA neuron firing rates vary across adolescence in male rats (McCutcheon and Marinelli, 2009; McCutcheon et al., 2012). We propose that all these cellular and molecular processes converge and interact, and that experience may impact DA development through any one - or multiple - pathways (Figure 6). Indeed, mild traumatic brain injury in adolescent rats induces sex-specific changes in Netrin-1 levels in the NAc (Kaukas et al., 2020), and also increases microglia-mediated pruning of DA receptors in this region (Cannella et al., 2020).

We are only at the beginning of understanding the complex interplay of genes and environmental factors that build DA circuitry in adolescence. Many interesting and important lines of inquiry remain to be addressed. The field will move forward by placing special emphasis on identifying molecular drivers of sex differences in mesocorticolimbic DA maturation and making the inclusion of male and female subjects obligatory in neurodevelopmental research. As work on these topics advances, a major focus should also be placed on unraveling epigenetic mechanisms linking adolescent experiences to changes in DA development, and on discovering non-invasive longitudinal biomarkers for evaluating the state of DA system development. This work would eventually allow for preventive and therapeutic interventions precisely targeted in time. MicroRNAs, for example, show promise to serve as such markers, as they regulate guidance cue genes in adolescence and are detectable in peripheral fluids (Torres-Berrío et al., 2020b; Morgunova and Flores, 2021). Finally, a critical question that remains open is whether and how “positive” experiences can promote healthy DA system development and improve mental health outcomes in emerging adults.

## Author Contributions

LMR and CF wrote the manuscript. All authors contributed to the article and approved the submitted version.

## Conflict of Interest

The authors declare that the research was conducted in the absence of any commercial or financial relationships that could be construed as a potential conflict of interest.

## Publisher’s Note

All claims expressed in this article are solely those of the authors and do not necessarily represent those of their affiliated organizations, or those of the publisher, the editors and the reviewers. Any product that may be evaluated in this article, or claim that may be made by its manufacturer, is not guaranteed or endorsed by the publisher.
